# Fumarase: From the TCA Cycle to DNA Damage Response and Tumor Suppression

**DOI:** 10.3389/fmolb.2018.00068

**Published:** 2018-07-25

**Authors:** Michael Leshets, Yardena B. H. Silas, Norbert Lehming, Ophry Pines

**Affiliations:** ^1^Department of Microbiology and Molecular Genetics, Institute for Medical Research Israel-Canada (IMRIC), Faculty of Medicine, Hebrew University of Jerusalem, Jerusalem, Israel; ^2^NUS-HUJ-CREATE Program and the Department of Microbiology and Immunology, Yong Loo Lin School of Medicine, National University of Singapore, Singapore, Singapore

**Keywords:** fumarase, DNA damage response, organic acids, mitochondria, protein dual targeting, tumor suppressor, metabolite signaling, DNA damage repair

## Abstract

Fumarase is an enzyme of the tricarboxylic acid (TCA) cycle in mitochondria, but in recent years, it has emerged as a participant in the response to DNA double strand breaks (DSBs) in the nucleus. In fact, this enzyme is dual-targeted and can be also readily detected in the mitochondrial and cytosolic/nuclear compartments of all the eukaryotic organisms examined. Intriguingly, this evolutionary conserved cytosolic population of fumarase, its enzymatic activity and the associated metabolite fumarate, are required for the cellular DNA damage response (DDR) to double-strand breaks. Here we review findings from yeast and human cells regarding how fumarase and fumarate may precisely participate in the DNA damage response. In yeast, cytosolic fumarase is involved in the homologous recombination (HR) repair pathway, through its function in the DSB resection process. One target of this regulation is the resection enzyme Sae2. In human cells, fumarase is involved in the non-homologous end joining (NHEJ) repair pathway. Fumarase is phosphorylated by the DNA-dependent protein kinase (DNA-PK) complex, which induces the recruitment of fumarase to the DSB and local generation of fumarate. Fumarate inhibits the lysine demethylase 2B (KDM2B), thereby facilitating the dimethylation of histone H3, which leads to the repair of the break by the NHEJ pathway. Finally, we discuss the question how fumarase may function as a tumor suppressor via its metabolite substrate fumarate. We offer a number of models which can explain an apparent contradiction regarding how fumarate absence/accumulation, as a function of subcellular location and stage can determine tumorigenesis. Fumarate, on the one hand, a positive regulator of genome stability (its absence supports genome instability and tumorigenesis) and, on the other hand, its accumulation drives angiogenesis and proliferation (thereby supporting tumor establishment).

## Introduction

The maintenance of genome integrity is one of the most important problems of all living organisms. An average human cell suffers approximately one hundred thousand different DNA lesions each day (Lindahl and Barnes, 2000; Alberts et al., 2004; Jackson and Bartek, 2009). Failure to repair the damaged DNA can lead to disease, the most prominent of which is cancer (Hanahan and Weinberg, 2011; O'Driscoll, 2012).

DNA double-strand breaks (DSBs) are one of the most cytotoxic damages that can be inflicted on our genetic material. Defective repair of these lesions can lead to gross chromosomal rearrangements, such as large deletions, translocations and insertions. Such rearrangements can lead to loss of tumor suppressor genes and oncogene misexpression, both of which have been implicated in cancer induction and progression (Lengauer et al., 1998; Richardson and Jasin, 2000; van Gent et al., 2001; Shiloh and Lehmann, 2004; Hanahan and Weinberg, 2011; O'Driscoll, 2012). Thus, identifying and characterizing unknown factors that play a role in the response to DSBs, is extremely important.

Different cellular mechanisms that repair DSBs have evolved during evolution (van Gent et al., 2001; Shiloh and Lehmann, 2004). In human and yeast cells there are two major pathways which are responsible for DSB repair; the first, non-homologous end joining (NHEJ), re-joins the DNA broken ends and is regulated in the yeast *Saccharomyces cerevisiae* by the yKu70/80 complex, Dnl4 and Lif1 (Feldmann and Winnacker, 1993; Boulton and Jackson, 1996; Feldmann et al., 1996; Mages et al., 1996; Schar et al., 1997; Teo and Jackson, 1997; Wilson et al., 1997; Herrmann et al., 1998; Ramos et al., 1998; Lewis and Resnick, 2000; Pracharoenwattana et al., 2010; Durdikova and Chovanec, 2017). In human cells this repair mechanism is induced by the DNA-dependent protein kinase (DNA-PK) complex, which is composed of the Ku70/80 heterodimer and the DNA-PK catalytic subunit (DNA-PKcs), and additional factors like the X-ray cross complementing protein 4 (XRCC4), DNA Ligase IV, XRCC4-like factor (XLF) and Aprataxin-and-PNK-like factor (APLF) (Mimori et al., 1986; Reeves et al., 1989; Yaneva et al., 1989; Paillard and Strauss, 1991; Higashiura et al., 1992; Gottlieb and Jackson, 1993; Li et al., 1995; Otevrel and Stamato, 1995; Wei et al., 1995; Critchlow et al., 1997; Grawunder et al., 1997; Ahnesorg et al., 2006; Buck et al., 2006; Bekker-Jensen et al., 2007; Iles et al., 2007; Kanno et al., 2007; Davis and Chen, 2013). The repair of DSBs using NHEJ is more error-prone and seldomly used in yeast, while in human cells this is the dominant repair pathway (Kramer et al., 1994; Moore and Haber, 1996; Lewis and Resnick, 2000; Shibata, 2017). The second DSB repair mechanism is homologous recombination (HR), in which an intact homologous DNA sequence is used to accurately repair the DSB (van Gent et al., 2001; Aylon and Kupiec, 2004; Shiloh and Lehmann, 2004). In order to repair the break by HR the DNA flanking the DSB, first must be exonucleolytically cleaved to form a 3′overhang structure in a process termed DSB resection. This process contains two sequential steps, initial resection which produces a short 3′ overhang region in the immediate vicinity of the DSB, and extensive resection that processively cleaves the 5′ strand to form a longer 3′ overhang structure. In yeast, the resection process is orchestrated by Mre11, Sae2, Exo1, and the Dna2-Sgs1/Top3/Rmi1 (STR) complex (White and Haber, 1990; Rattray et al., 2001; Clerici et al., 2005; Mimitou and Symington, 2008; Zhu et al., 2008; Cannavo and Cejka, 2014). In human cells, the DSB resection is performed by MRE11, C-terminal binding protein interacting protein (CtIP), EXO1 and the DNA2-Bloom helicase (BLM) complex (Sartori et al., 2007; Buis et al., 2008; Nimonkar et al., 2011; Zhou et al., 2014; Anand et al., 2016).

## Fumarase, its canonical function and subcellular locations

Fumarase is a member of the class II fumarase enzymes which is conserved from prokaryotes to humans. In the yeast, *S. cerevisiae*, the enzyme fumarase is encoded by the *FUM1* gene whose product is a homotetramer with a molecular weight of about 200 kDa (Wu and Tzagoloff, 1987; Woods et al., 1988; Burak et al., 2013). Fumarase catalyzes the hydration of fumarate to *L*-malate and the reverse dehydration reaction (Mann and Woolf, 1930; Woods et al., 1988). Fumarase is found in mitochondria where it participates in the tricarboxylic acid (TCA) cycle.

**Figure 1:**
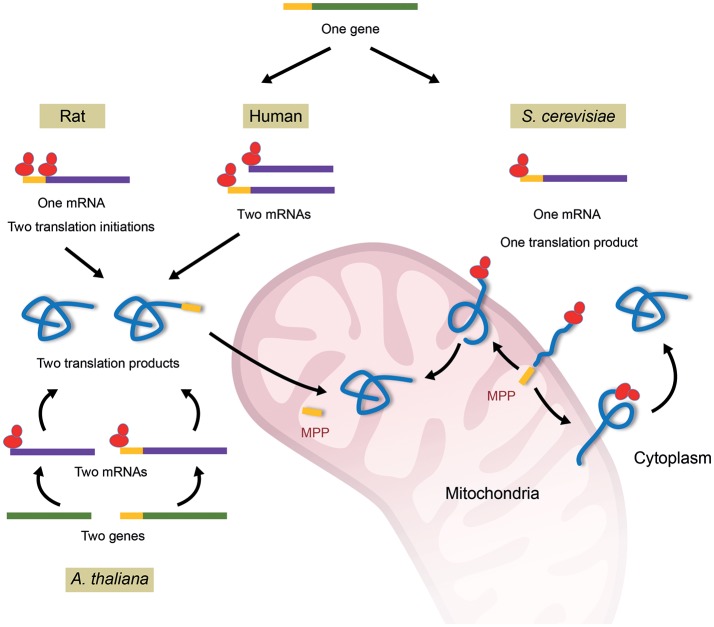
Mechanisms of fumarase dual targeting in different organisms. In ***S. cerevisiae*** all fumarase molecules are first targeted to mitochondria, begin their translocation and are processed by the mitochondrial processing peptidase (MPP). Some of the molecules move back to the cytosol in a process termed “reverse translocation”; If folding of the fumarase protein molecule starts in mitochondria it will be localized to the mitochondrial matrix, however, if folding of the protein molecule starts outside mitochondria it will reside in the cytosol. In other words, the folding of fumarase is the driving force for its localization. In **human**, a single fumarase gene encodes two groups of mRNAs either encoding a full-length mitochondrial precursor that harbors an MTS, or a shorter cytoplasmic polypeptide that lacks it. In **rat**, a single fumarase gene encodes a single mRNA, which by differential translation initiation produces a full-length mitochondrial precursor that harbors an MTS and a shorter cytoplasmic polypeptide that lacks it. ***A. thaliana*** harbors two highly homologs fumarase genes that encode a mitochondrial or cytosolic protein, either containing or lacking an MTS respectively. Sequences encoding or indicating the mature fumarases are in green lines for DNA, purple for mRNA and light blue for protein. The MTS sequences are indicated by yellow lines and ribosomes colored red.

In addition to the mitochondrial fumarase, the enzyme can also be found in the cytosolic compartment. The cytosolic localization of fumarase is highly conserved, as the enzyme can be found in the cytosol of most eukaryotes extending from yeast to human (Tolley and Craig, 1975; Edwards and Hopkinson, 1979; Kobayashi and Tuboi, 1983; Akiba et al., 1984; O'Hare and Doonan, 1985; Wu and Tzagoloff, 1987). These dual localized proteins are coined “echoforms,” indicating repetitious forms of the same protein distinctly placed in the cell. There are a number of known mechanisms that regulate the subcellular distribution of fumarase in eukaryotes (Figure 1). In *S. cerevisiae*, both cytosolic and mitochondrial fumarase echoforms are encoded by the *FUM1* gene (Wu and Tzagoloff, 1987). In the course of translation, a subset of *FUM1* translation products, which are partially translocated, fold outside mitochondria and are blocked for full mitochondrial import by a mechanism termed reverse translocation (Figure 1, right, *S. cerevisiae*). Upon translation termination, these folded translation products remain in the cytosol constituting the cytosolic fumarase population (Stein et al., 1994; Sass et al., 2001, 2003; Yogev and Pines, 2011; Kalderon and Pines, 2014). In human cells, the human homolog of fumarase, termed fumarate hydratase (FH), is expressed from a single gene (Figure 1, top middle) (van Someren et al., 1974; Craig et al., 1976). The fumarase gene promoter was shown to contain multiple transcription start sites from which two groups of fumarase mRNAs are transcribed. The first group includes transcripts, which are translated into proteins that contain the fumarase mitochondrial targeting sequence (MTS), while the second group translates into fumarase proteins which lack this sequence. Following translation, these two versions of the protein constitute the mitochondrial and cytosolic echoforms of fumarase, respectively (Dik et al., 2016). In rat liver, it has been suggested that the two translation products (as above in human), one containing and one lacking the MTS, are formed by alternative translation initiation (Figure 1, top left) (Suzuki et al., 1989; Tuboi et al., 1990). This same situation of two translation products is achieved in *Arabidopsis thaliana* by two nearly identical genes, one that encodes fumarase with an MTS and one that lacks the MTS (Figure 1, bottom) (Pracharoenwattana et al., 2010).

## Cytosolic fumarase plays a role in the DNA damage response (DDR) to DNA double strand breaks (DSBs)

With the canonical role of fumarase in the TCA cycle and mitochondria, it was unclear what the function of the enzyme in the cytosol is. To address the question of the cytosolic fumarase function in *S. cerevisiae*, Yogev *et al*. constructed a strain termed Fum^M^. The *FUM1* gene in this strain was deleted from its original location on chromosome 16 and inserted into the mitochondrial DNA. This resulted in the depletion of cytosolic fumarase, while the mitochondrial population of the enzyme was retained, thus presenting an opportunity to determine the cytosolic function of fumarase (Yogev et al., 2010).

The Fum^M^ strain exhibited significant sensitivity to HO-induced DSBs, γ-irradiation and DSB-inducing chemicals. As a consequence of DSB induction in wild type (WT) yeast, fumarase expression levels increased and the enzyme was now also found in the cell nucleus. Expression of cytosolic fumarase or exposure of the cells to fumarate, suppressed the DSB sensitivity of the Fum^M^ strain (Yogev et al., 2010). We conclude that the enzymatic activity of cytosolic fumarase is important for the DNA damage response (DDR) to DSBs.

**Figure 2:**
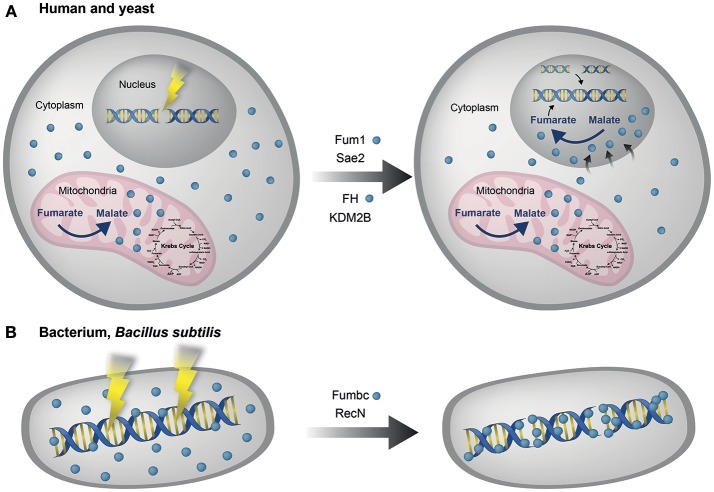
Fumarase is involved in the eukaryotic and prokaryotic DNA damage response. **(A) Human and yeast**. Fumarase is a TCA cycle enzyme which catalyzes the conversion of fumarate to L-malate in the mitochondria. Upon DNA damage the cytosolic echoform of fumarase is localized to the nucleus, there, its enzymatic activity catalyzes the reverse conversion of malate to fumarate, so causing local accumulation of fumarate. This accumulation of fumarate (by fumarase) is required for the proper function of the DNA damage response (DDR) to double strand breaks (DSBs), in both human (FH) and yeast (Fum1) cells via targets such as KDM2B and Sae2 respectively. **(B) Bacterium**, *Bacillus subtilis*. Fumarase of *Bacillus subtilis*. (Fumbc) is also a TCA cycle enzyme and is induced upon DNA damage. Fumbc is co-localized with the bacterial DNA. Fumbc dependent intracellular signaling of the *B. subtilis* DNA damage response is achieved via production of L-malate, which affects the translation of RecN, the first protein recruited to DNA damage sites (Singer et al., 2017). Blue circles indicate fumarase in the different organisms (human, yeast, and bacteria).

Yogev et al. also showed that fumarase is required for the DSB DDR in human cell lines. Following DSB induction the cellular levels of fumarase increased and localization of the protein to the nucleus was observed. In addition, fumarase knockdown has been shown to increase cell susceptibility to ionizing radiation and hydroxyurea (HU) induced DSBs (Yogev et al., 2010). These results from human and yeast cells were the basis for our original model of fumarase function in the DDR (Figure 2A). Worth mentioning here, as will be referred to in the “Concluding remarks,” is the finding that a bacterial fumarase (of *Bacillus subtilis*, Fum-bc) is induced upon DNA damage, co-localized with the bacterial DNA and participates in the DDR (Figure 2B). Thus, the dual function of fumarase in the TCA cycle and the DDR may be an ancient feature of prokaryotes and eukaryotes.

## Yeast fumarase is involved in DSB resection

**Figure 3:**
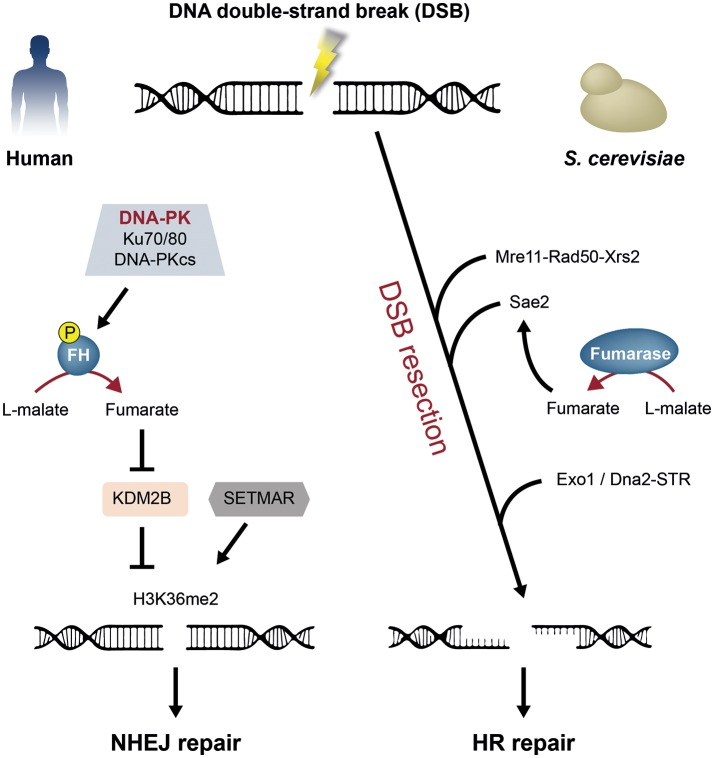
Fumarase functions in the human and yeast DNA damage response (DDR) to double-strand breaks (DSBs). Two DSB repair pathways in human and yeast are non-homologous end joining (NHEJ) and homologous recombination (HR). **Left panel:** Upon DSB formation in human cells, fumarase (FH) is phosphorylated on Thr236 by the DNA-dependent protein kinase (DNA-PK) complex. This modification induces the recruitment of fumarase to the DSB and local generation of fumarate. Fumarate inhibits the lysine demethylase 2B (KDM2B), thereby facilitating the dimethylation of histone H3 on lysine 36 (H3K36me2) by the SET domain and mariner transposase fusion protein (SETMAR). This leads to the repair of the break by the NHEJ pathway. **Right panel:** The repair of a DSB by the HR pathway, requires that the DNA flanking the DSB undergoes resection. In yeast, the resection process is orchestrated by the Mre11-Rad50-Xrs2 complex, Sae2, Exo1, and the Dna2-STR complex. The yeast cytosolic fumarase and the metabolite fumarate affect the DSB resection process, by regulating the protein level of the resection factor Sae2.

Leshets et al., have found that yeast cytosolic fumarase is important for the HR repair pathway, through its function in the initial step of the DSB resection process (Figure 3, right) (Leshets et al., 2018). Supporting this notion is that no genetic interactions were detected with the extensive resection factors Exo1 and Sgs1 (Leshets et al., 2018). Moreover, previous publications indicated that during the initial step of resection 50 to 1,600 bases of DSB flanking DNA can be processed (Mimitou and Symington, 2008; Zhu et al., 2008; Garcia et al., 2011). In that study the resection assay measures resection 0.29 kbp upstream the HO cut site. If following the depletion of cytosolic fumarase only the extensive step of resection is affected, one would detect at least some level of initial resection 0.29 kbp from the DSB. In fact, resection was not detected, suggesting that cytosolic fumarase is involved in the initial step of the DSB resection process (Leshets et al., 2018).

The functional interaction between Sae2 and cytosolic fumarase further supports the role of fumarase in the initial step of resection. The interaction was first suggested by the similar phenotypes of the Fum^M^ and the Δ*sae2* strains. Both strains exhibit postponed dissociation of Mre11 from DSBs, decreased resection and impaired kinetics of DSB repair (Lisby et al., 2004; Clerici et al., 2005; Ferrari et al., 2015). The DSB susceptibility of the Fum^M^ strain was partially suppressed by overexpression of Sae2 and this reconstituted its resection capacity. A split-ubiquitin assay indicated that these proteins physically interact *in vivo*, and a direct interaction *in vitro* was shown by a column retention assay. We still did not know whether cytosolic fumarase acts upstream of Sae2, and if so, how does it regulate this endonuclease? One hint was the reduced protein levels of Sae2 in cytosolic fumarase depleted cells, suggesting that fumarase acts upstream of Sae2, which we presume is regulated by determining its protein abundance. In this regard, cytosolic fumarase regulation of Sae2 is at the protein level, and not at the Sae2 mRNA level (Leshets et al., 2018). It is possible that cytosolic fumarase may enhance the translation of Sae2 or have a negative effect on its degradation.

Mre11 nuclease activity has been shown to be part of the initial step in the resection process, both in human, and yeast cells (Cannavo and Cejka, 2014; Anand et al., 2016). Exposure to the metabolite fumarate can inhibit the DSB sensitivity of the Mre11 nuclease dead (*mre11-nd*) mutant cells (Leshets et al., 2018), suggesting that fumarate is involved in the resection process. Nevertheless, how fumarate affects Sae2 is still unclear.

## Does yeast cytosolic fumarase have additional roles in the DSB DDR pathway?

We assume that cytosolic fumarase may be important for the DDR not only through its functional relationship with Sae2. Supporting this assumption is the fact that fumarase and Sae2 are not epistatic (Leshets, thesis 2018). It has been previously shown that the depletion of Sae2 only partially impairs the resection process (Clerici et al., 2005; Ferrari et al., 2015). In comparison, the inhibition of resection is much more profound upon cytosolic fumarase depletion (Leshets et al., 2018). These observations suggest that cytosolic fumarase may be involved with additional resection factors. In this regard, no genetic interactions have been detected with Exo1 or Sgs1 (Leshets et al., 2018).

## Human fumarase and the NHEJ pathway

A consequence of DSB formation, is the phosphorylation of fumarase on Thr 236 by the DNA-dependent protein kinase (DNA-PK) (Jiang et al., 2015). This phosphorylation is required for the recruitment of fumarase to the DSB. Following its recruitment, fumarase-mediated fumarate production inhibits the α-ketoglutarate-dependent lysine demethylase 2B (KDM2B). KDM2B inhibition increases the histone H3 dimethylation on lysine 36 (H3K36me2) which leads to accumulation of the DNA-PK complex and subsequent repair of the break by NHEJ (Figure 3, left). The phosphorylation of histone H2AX (γ-H2AX) is a central event during DSB DDR. One of the kinases, which was shown to induce H2AX phosphorylation, is the DNA-PK complex. Interestingly, the mutation of Thr 236 of fumarase does not affect γ-H2AX levels, even though it is expected to do so due to the reduced DNA-PK accumulation at the DSB (Stiff et al., 2004; An et al., 2010; Jiang et al., 2015). The aberrant kinetics of H2AX phosphorylation because of the fumarase knockdown was previously described by Yogev et al. and confirmed by Jiang et al. Nevertheless, the Thr 236 mutation did not affect γ-H2AX (Yogev et al., 2010; Jiang et al., 2015). This observation suggests that fumarase's role in the DSB DDR is not restricted to the NHEJ pathway (e.g., HR as in yeast).

## Is human fumarase also involved in the HR pathway?

A functional relationship between fumarase and α-ketoglutarate-dependent histone demethylases is very intriguing due to the emerging importance of histone methylation for the DDR. Indeed, fumarase has been shown to influence the global histone methylation pattern and fumarate was shown to inhibit several members of the KDM family, including KDM4A (Xiao et al., 2012). KDM4A is a tri-methylase capable of converting H3K36me3 to H3K36me2, while SET domain-containing protein 2 (Setd2) methyltransferase is responsible for the generation of H3K36me3 (Whetstine et al., 2006; Edmunds et al., 2008). These observations suggest that fumarase-dependent fumarate production may inhibit KDM4A, thus facilitating the generation of H3K36me3 by Setd2. This histone modification is especially intriguing due to the fact that H3K36me3 has been shown to be important for the repair of DSBs by the HR pathway (Carvalho et al., 2014; Pfister et al., 2014). This is supported by the observation that transcriptionally active chromatin which is marked by H3K36me3, is preferentially repaired by HR (Aymard et al., 2014).

It has been proposed that H3K36me3 is important for the HR repair due to its involvement in the DSB resection process. Two comprehensive studies proposed that Setd2-dependent H3K36me3 is required for recruitment of the lens epithelium-derived growth factor p75 splice variant (LEDGF) to the chromatin. Following DSB induction, LEDGF has been shown to recruit CtIP which facilitates the initiation of resection (Sartori et al., 2007; Daugaard et al., 2012; Pfister et al., 2014; Anand et al., 2016). In concert, these results may imply that fumarase facilitated H3K36 tri-methylation can induce DSB resection, thus committing the cell to the repair of the DSB by HR.

The deduction above and the results presented by Jiang et al. propose a complex model of fumarase involvement in the response to DSBs in human cells. Fumarase mediated fumarate production may facilitate the generation of H3K36me2 or H3K36me3 by the inhibition of KDM2B or KDM4A, respectively (Xiao et al., 2012; Jiang et al., 2015). These suggested capacities of fumarase support its importance in both NHEJ and HR repair pathways.

## Human fumarase functions as a tumor suppressor

Fumarase was shown to be a tumor suppressor. Heterozygous mutations in the fumarase gene are associated with hereditary leiomyomatosis and renal cell cancer (HLRCC) syndrome. Patients with HLRCC can suffer from multiple uterine and cutaneous leiomyomas and tend to develop type II papillary renal cell carcinoma. HLRCC is a syndrome which is dominantly inherited and is considered a two-hit condition. Essentially all of the HLRCC tumors of patients exhibit inactivation of both fumarase alleles. These findings emphasize that the complete loss of fumarase activity is required for the tumorigenesis process (Reed et al., 1973; Kiuru et al., 2001; Launonen et al., 2001; Tomlinson et al., 2002). While there is well-known involvement of fumarase in HLRCC, mutations in it are rarely detected in sporadic tumors. Nonetheless, biallelic inactivation of fumarase has been reported in some cases of uterine leiomyomas, soft tissue sarcoma, and type II papillary renal cell carcinomas (Barker et al., 2002; Kiuru et al., 2002; Lehtonen et al., 2004; Gardie et al., 2011).

Much effort has been put into determining the mechanism by which fumarase functions as a tumor suppressor. The sole leading model for some years was that the loss of fumarase activity and the buildup of fumarate concentrations inhibits PHD 1, 2 and 3, which are α-ketoglutarate-dependent prolyl hydroxylase enzymes. PHD inhibition stabilizes the α subunit of HIF (hypoxia-inducible transcription factor), which leads to the establishment of an active HIF transcription complex. High levels of HIF have been shown to enhance angiogenesis and glucose metabolism, both of which are known to be essential for tumorigenesis (Isaacs et al., 2005; Pollard et al., 2005; Selak et al., 2005; Vanharanta et al., 2006; Hanahan and Weinberg, 2011). Nevertheless, the recent data discussed above suggest that fumarase is also important in order to maintain genomic stability (Yogev et al., 2010; Jiang et al., 2015). According to this scenario, the loss of fumarase, as a guardian of genome integrity, can also contribute to the development of cancer.

## The paradoxical nature of the mechanism by which fumarase acts as a tumor suppressor

There are two proposed models for the activity of fumarase as a tumor suppressor. In the first, the loss of fumarase is suggested to block the TCA cycle in mitochondria causing the accumulation of fumarate, which subsequently leads to the stabilization of HIF (Isaacs et al., 2005; Pollard et al., 2005; Selak et al., 2005; Vanharanta et al., 2006). The second model, suggests that the loss of fumarase diminishes the ability of the cell to generate fumarate, thereby compromising genomic stability (Yogev et al., 2010; Jiang et al., 2015). In both models, fumarate is the key effector molecule, but in the first model it induces an oncogenic effect, while in the second, fumarate acts as an inhibitor of oncogenicity. Considering both models, the problem is that inactivation of fumarase in a cell is proposed to lead to both accumulation of fumarate, due to the blockage of the TCA cycle, and also to the inability to generate fumarate for the DDR. This discrepancy raises the question how can these two apparently contradictory models be reconciled.

**Figure 4:**
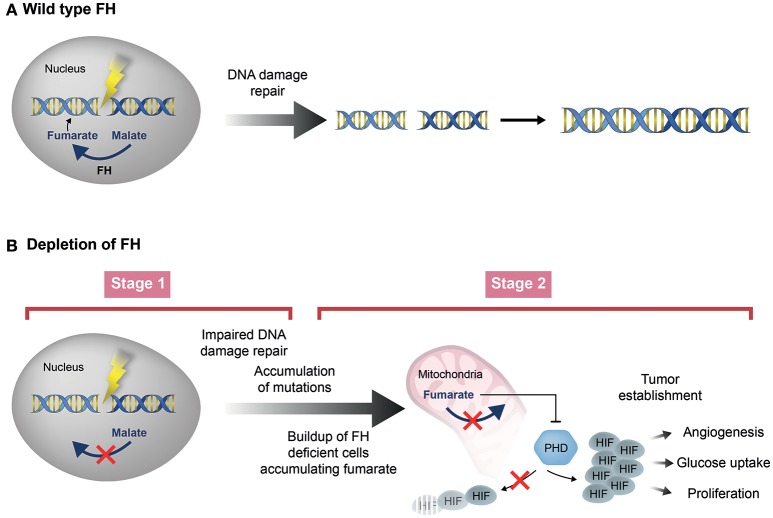
Two stage model of fumarase depletion leading to tumorigenesis. **(A)** Fumarase and its metabolite fumarate are involved in the DNA damage response in human cells. **(B)** Depletion of fumarase can contribute to the tumorigenesis process. In the first stage (Stage 1), biallelic inactivation of fumarase in a single tumor cell may abolish the ability of the cell to generate fumarate in the proximity of the cellular DNA, thereby decreasing genomic stability, leading to creation of additional mutations. At this stage, the fumarate concentration in the single tumor cell may not be sufficient for HIF stabilization. In the second stage (Stage 2), proliferation of the fumarase deficient cells may form a closely positioned cell population, in which the fumarate levels rise and the concentration required for HIF stabilization can be achieved.

The first plausible answer to this question may rely on the possibility that in order to function in the DDR, fumarase must generate high concentrations of fumarate near the cellular DNA, in proximity of the DSB (Figure 4A). This possibility is supported by the fact that upon DSB induction fumarase was shown to localize to the cell nucleus and even form nuclear foci (Yogev et al., 2010; Jiang et al., 2015). Considering this, it is plausible that upon biallelic inactivation of the enzyme, the increase in the cellular concentration of fumarate is sufficient for the stabilization of HIF, but not high enough to compensate for the lack of fumarase near the cellular DNA. The second possibility which, is an extension of the first, argues that the two mechanisms by which fumarase acts as a tumor suppressor, occur at different stages of tumor development (Figure 4B). In the first stage, biallelic inactivation of fumarase in a single tumor cell may abolish the ability of the cell to generate fumarate in the proximity of the cellular DNA, thereby decreasing genomic stability. Nevertheless, at this stage the fumarate concentration in the single tumor cell may not be sufficient for HIF stabilization. In the second stage, proliferation of the fumarase deficient cells may form a closely positioned cell population, in which the fumarate concentration required for HIF stabilization can be achieved. According to this scenario, the loss of fumarase first reduces the genomic stability of the cell, and only later causes HIF stabilization (Figure 4B). Unfortunately, while fumarate levels have been determined in established FH-deficient tumors, there is no data regarding the levels of this metabolite at stages in which the loss of FH occurs. Until such measurements of fumarate levels at different stages of tumor development are available, the second scenario above, although plausible, remains speculative.

## Concluding remarks

Fumarase is a highly conserved metabolic enzyme of the TCA cycle which is involved in the two main DSB repair pathways in eukaryotes; NHEJ in human and HR in yeast. The enzyme and its associated metabolite fumarate, interact and affect different components of the DDR pathways (e.g., KDM2B, Sae2, Figure 2A, 3). To this setting we can add a recent study by Singer *et al*. which shows that fumarase in prokaryotes already possessed both TCA cycle and DDR functions (Singer et al., 2017). Fumarase of *Bacillus subtilis* (Fum-bc) a prokaryote bacterium is induced upon DNA damage, co-localized with the bacterial DNA and participates in the DDR (Figure 2B). Intriguingly, Fum-bc can complement both eukaryotic functions (TCA cycle and DDR) when expressed in yeast. Fumarase dependent intracellular signaling of the *B. subtilis* DDR is achieved via production of *L*-malic acid, which affects the translation of RecN, the first protein recruited to DNA damage sites (Singer et al., 2017). Thus, different fumarase related metabolites function in the DDR of different organisms. One take home message is that it is the fumarase related metabolites which are the active molecules in the DDR, but they must be administered at specific locations in the cell and that is why the enzyme that produces these molecules must be localized to the right place in the cell.

With respect to evolution, it appears that for fumarase, the two functions came first, already in the prokaryote, thereby creating the driving force for dual localization of the protein in the eukaryotic cell. The notion that during evolution, cellular functions such as the DDR can recruit different primary metabolite signaling molecules is exciting.

Recent studies have extended our understanding of the possible functions of fumarase in the DDR. Nevertheless, additional enquiries are needed in order to decipher the complex role of this enzyme and its associated metabolites in the different DDR pathways. Deeper comprehension of this role will help us fully understand the function of fumarase in health and disease, and in particular its functions as a tumor suppressor (Figure 4).

## Author contributions

ML and YS are Ph.D. students, NL is a collaborator, OP is an expert on protein dual targeting and dual function, in particular regarding the enzyme fumarase.

### Conflict of interest statement

The authors declare that the research was conducted in the absence of any commercial or financial relationships that could be construed as a potential conflict of interest.
